# Purine bias in bacterial genes is driven by runaway transcription

**DOI:** 10.1038/s41564-026-02389-1

**Published:** 2026-06-15

**Authors:** K. Julia Dierksheide, James C. Taggart, Grace E. Johnson, Gene-Wei Li

**Affiliations:** 1 Department of Biology, Massachusetts Institute of Technology, Cambridge, MA USA; 2 Howard Hughes Medical Institute, Cambridge, MA USA; 3 Present Address: Department of Systems Biology, Harvard Medical School, Boston, MA USA; 4 Present Address: Department of Molecular Biology, Princeton University, Princeton, NJ USA

**Keywords:** Bacterial transcription, Gene regulation, Bacterial genomics, Transcription, Bacterial systems biology

## Abstract

Genes in many bacteria are rich in purine nucleotides (A and G), but the origin of this preference is unclear. Here, using a large-scale reporter assay in *Bacillus subtilis*, we show that this purine bias is critical for gene expression. It prevents premature transcription termination in species that exhibit runaway transcription, in which RNA polymerases outpace ribosomes, leaving nascent mRNA exposed to the termination factor Rho. This vulnerability is resolved by a divergence between Rho’s heightened target specificity and coding-strand nucleotide content. Selective pressure to avoid Rho appears to drive strong gene purine bias across species that exhibit runaway transcription, except in lineages that have lost Rho. This purine requirement underlies codon usage biases, promotes suppression of pyrimidine-rich antisense transcripts and can suppress expression of engineered constructs. Our results suggest that avoidance of premature transcription termination imposes major constraints on nucleotide content during genome evolution and adaptation of foreign genes.

## Abstract (web summary)

Purine bias is critical for gene expression in bacteria, preventing premature transcription in species that exhibit runaway transcription.

## Main

Protein production requires complete synthesis of mRNA molecules^1^. In the model bacterium *Escherichia coli*, premature termination of mRNA transcription is inhibited by a closely trailing ribosome, which prevents nascent RNAs from triggering either intrinsic or Rho-dependent termination^2–9^. However, in other bacteria, transcription is not always tightly coupled to translation: RNA polymerases (RNAPs) outpace ribosomes in many gram-positive bacteria with low guanine + cytosine (GC) content, such as *Bacillus subtilis*^10,11^. It remains unclear what alternative mechanisms broadly protect these ‘runaway’ RNAPs from premature termination.

In particular, runaway RNAPs are vulnerable to the conserved transcription termination factor Rho. In bacteria, Rho is thought to function as a global surveillance factor by terminating untranslated RNAs^6,12–15^—inhibition of Rho in both *E. coli* and *B. subtilis* dramatically elevates antisense, but not sense, transcription^16–19^. In *E. coli*, Rho recognizes a series of up to six cytosine–cytosine (CC) or uracil–cytosine (UC) dinucleotides interspersed on nascent RNA^20–23^. This signal is degenerate enough that many RNAs can be targeted in the absence of a closely trailing ribosome^2,4,5,13–15,24–27^. Indeed, in the classic examples of polarity, premature stop codons frequently result in Rho-dependent termination of downstream transcription^6,28–32^. However, for species in which runaway RNAPs expose nascent mRNAs to Rho, this promiscuous targeting model would predict widespread termination and is not compatible with the observation of selective termination of antisense transcription.

On the basis of an observation that *B. subtilis* genes are particularly rich in purines (adenine (A) and G), complementing the degenerate pyrimidine preference (C and U) of *E. coli* Rho, we hypothesized that this biased sequence composition, rather than translation state, may form the basis for Rho’s selective avoidance of mRNA in *B. subtilis*. Using a large-scale reporter library to identify regions of transcription termination across the *B. subtilis* genome, we show that sequence composition alone allows *B. subtilis* genes to escape Rho termination. In contrast to *E. coli* Rho activity, *B. subtilis* Rho activity was found to be highly sequence specific, terminating the transcription of only a small set of genomic fragments strongly biased towards antisense regions with high C and U content. Avoidance of such pyrimidine-rich regions appears to broadly drive purine bias in genes and constrain codon usage across Bacilli that encode Rho. This global coding constraint not only shapes gene evolution within individual species but also influences foreign gene expression. More broadly, our study highlights the outsized influence of a single transcription factor on the sequence of genes, cracking a hidden code within bacterial genomes.

## Results

### Genome-wide mapping of Rho termination

**Fig. 1:**
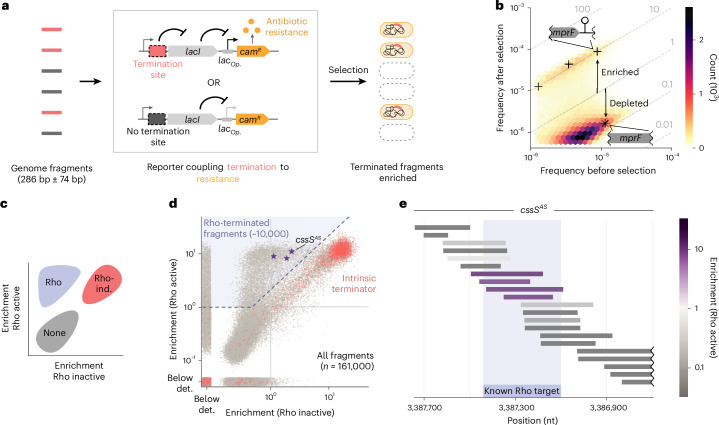
Genome-wide screen for sequences subject to Rho-dependent termination. **a**, Schematic of growth-based massively parallel reporter assay for termination activity. Left: genomic DNA fragments containing (pink) and lacking (grey) transcription termination signals. Mean fragment length was 286 bp with a standard deviation of 74 bp. Middle: reporter coupling termination to antibiotic resistance. *lacI*, lac repressor gene; *lac*_*Op.*_, lac operator; *cam*^*R*^, chloramphenicol resistance gene; orange circles, protein conferring antibiotic resistance. Selection refers to growth in chloramphenicol. Right: dead (white) and resistant (orange) bacterial cells. **b**, Distribution of fragment frequencies before versus after selection (Rho active; BCM-Bz absent). Grey dashed lines mark enrichment values. Daggers indicate fragments that contain the intrinsic terminator following *mprF*^32^. The asterisk indicates a fragment completely overlapping with the *mrpF* coding strand. **c**, Schematic indicating expected positions of fragments with no termination (None), Rho-dependent termination (Rho) or Rho-independent termination (Rho-ind.) activity in the plot shown in **d**. **d**, Enrichment of each fragment in the presence (Rho inactive) versus absence (Rho active) of the Rho inhibitor BCM-Bz. Rho-terminated fragments are defined as those in the region above the dashed lines, shaded in purple (Methods). Purple stars indicate putative Rho-terminated fragments antisense to *cssS*. Fragments containing strong intrinsic terminators (wild-type termination efficiency ≥75% (ref. ^32^)) are shown in pink. Below det., fragment below the detection limit after selection (Methods). **e**, Enrichment of fragments tiling the genomic region antisense to *cssS*. The segment previously shown to induce Rho termination^8^ is indicated by the purple-shaded region. Position indicates the genomic coordinate. Fragments extending beyond plot boundaries are cut off by a zigzag line. Source data

If sequence composition protects mRNA from Rho termination in *B. subtilis*, then isolated, untranslated fragments of coding sequence should not be terminated by Rho. To test this hypothesis, we designed a massively parallel reporter assay to measure Rho termination during transcription of 10^5^ DNA fragments (mean length = 286 base pairs (bp), s.d. = 74 bp) tiling the *B. subtilis* genome (5.4-fold coverage over 98% of the genome). We linked transcription termination to chloramphenicol resistance via a transcriptional reporter library in which DNA fragments are placed upstream of *lacI*, which represses expression of the resistance gene (Fig. 1a). Termination within a fragment reduces *lacI* expression, activating resistance and allowing growth in chloramphenicol. After chloramphenicol selection of the pooled reporter library, variant enrichment is quantified using deep DNA sequencing, revealing fragments that carry termination signals. Rho-dependent termination events were identified by a loss of enrichment when the selection was carried out in a separate culture containing the Rho inhibitor bicyclomycin-benzoate (BCM-Bz)^33^ (Methods).

**Extended Data Fig. 1:**
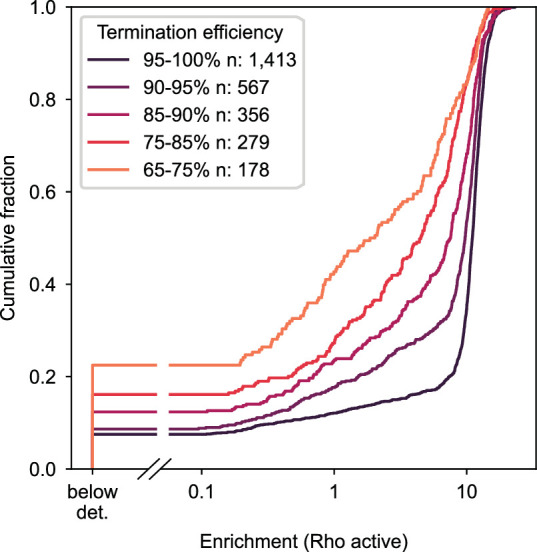
Fragment enrichment is correlated with termination efficiency. Related to Fig. 1. Cumulative distribution of enrichment in the absence of BCM-Bz (Rho active) for fragments containing an annotated intrinsic terminator, separated by termination efficiency^34^ (Methods). Fragments with more than 100 reads in the pre-selection sample are included. *n* indicates number of fragments in each distribution. Below det., below detection limit (Methods). Source data

Our massively parallel assay successfully distinguished genomic fragments that contain termination signals. While most fragments were depleted after chloramphenicol treatment, a distinct subpopulation (14%) was enriched by more than tenfold. This subpopulation includes most annotated intrinsic terminators^34^ (Fig. 1b–d), whose enrichment values were correlated with previously estimated efficiencies, ranging from 65% to 99% termination (Extended Data Fig. 1).

**Fig. 2:**
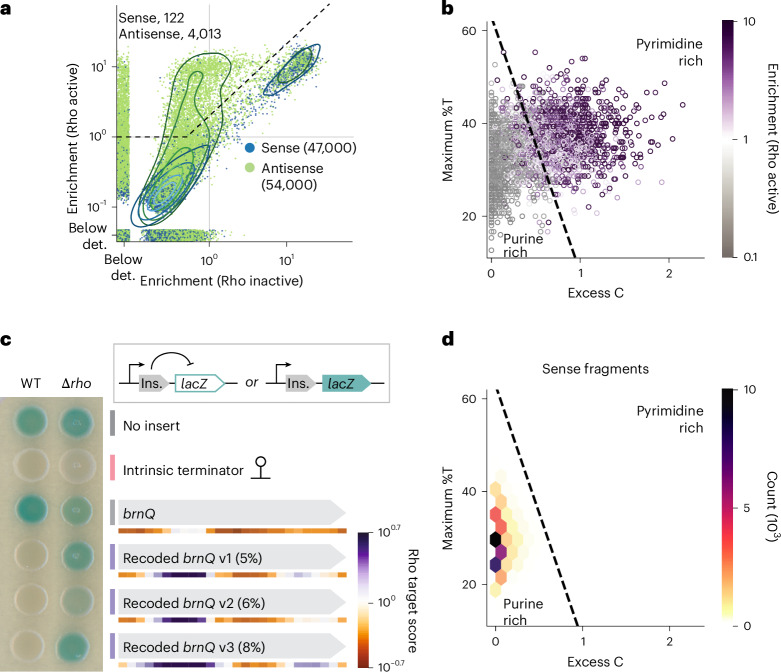
*B. subtilis* Rho terminates pyrimidine-rich antisense sequences. **a**, As in Fig. 1d, but for fragments that fully overlap with the sense (blue) or antisense (green) strand of annotated genes. The legend indicates the total number of each fragment type. Top left: count of each fragment type in Rho-terminated fragments. Contour lines mark the 10th, 25th, 50th and 75th percentiles of Gaussian kernel density estimates, excluding points below detection. **b**, Sequence features of 1,500 non-terminated fragments (>500 reads before selection) and 1,500 Rho-terminated fragments coloured by enrichment in the absence of BCM-Bz (fragment length ≥150 bp). Excess C quantifies C content relative to G content. Maximum %T, maximum T content in 150-nt windows tiling the sequence (Methods). The dashed line indicates the predicted boundary for Rho-terminated sequences (Rho target score = 10^0^) (Methods). **c**, β-galactosidase (LacZ) assays for termination of recoded *brnQ* variants in wild-type (WT) and Δ*rho* backgrounds. Top right: reporter schematic. Ins., insert. Left: image of *B. subtilis* reporter strains with the indicated inserts spotted on an X-gal plate (Methods). The percentage of nucleotides substituted is indicated in parentheses (insert sequences are available in Supplementary Table 3). Heat maps below each *brnQ* sequence show the Rho target score in 300-nt sliding windows (Methods). Coloured lines summarize the results: grey, no termination; purple, Rho termination; and pink, Rho-independent termination. Uncropped plate image in Extended Data Fig. 5a. **d**, Distribution of sequence features of the sense fragments shown in **a**, excluding fragments shorter than 150 nt (*n* = 46,777 fragments) (Methods). Dashed line as in **b**. Source data

Nearly half of the enriched fragments in the library were terminated by Rho, as evidenced by their sensitivity to BCM-Bz (Fig. 1c,d and Methods). This subset of ~10,000 fragments not only captured known targets of Rho termination but also resolved the sites of termination given the tiling nature of the library (Fig. 1d,e). Notably, Rho-terminated fragments are heavily depleted of coding-strand sequences (1% fully overlap with genes versus 29% in the starting library; Fig. 2a), supporting our hypothesis that Rho termination signals are broadly excluded from mRNA sequences in *B. subtilis*. The Rho-terminated fragments found in coding sequences (1%) are also substantially depleted when compared with those that trigger Rho’s helicase activity (>10% in coding sequences^35^), suggesting that the specificity is provided in additional steps in the termination pathway. Overall, this *cis*-encoded selectivity contrasts sharply with what has been observed for *E. coli* Rho, which often terminates transcription of coding regions when translation is inhibited^2,4–6,13–15,24,26,28–32^.

**Extended Data Fig. 2:**
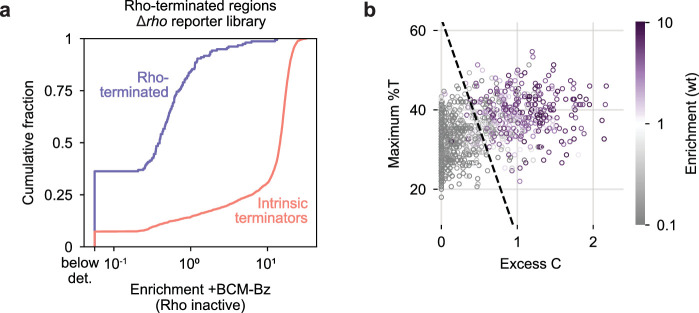
Similar Rho termination features are identified with BCM-Bz treatment and in the absence of Rho. Related to Figs. 1, 2. **a**, Distribution of enrichment in the presence of BCM-Bz for fragments in the BCM-Bz library overlapping with sites of Rho-dependent termination activity identified in the Δ*rho* screen (purple line, n = 157) and fragments in the BCM-Bz library containing intrinsic terminators (pink line, n = 3,037) (Methods). Below det., below detection limit (Methods). **b**, Sequence features of 630 nonterminated fragments ( > 100 reads pre-selection) and 348 Rho-terminated fragments from Δ*rho* screen colored by enrichment in wild-type background (fragment length ≥ 150 bp and ≤ 525 bp). Excess C quantifies C content relative to G content. Maximum %T is maximum T content in 150 nt windows tiling the sequence (Methods). Dashed line as in Fig. 2b. Source data

To validate the results from BCM-Bz treatment, we performed a smaller-scale selection assay in a strain without Rho (Methods). Consistent with strong Rho inhibition by BCM-Bz, Rho termination sites identified in this selection were broadly depleted during BCM-Bz treatment (Extended Data Fig. 2a), and these fragments also exhibited a strong depletion of coding-strand sequences (Supplementary Table 1). Together, these results indicate that BCM-Bz drove near-complete Rho inhibition in our assay, allowing for accurate identification of Rho-dependent termination events.

### Specificity of Rho-dependent termination

**Extended Data Fig. 3:**
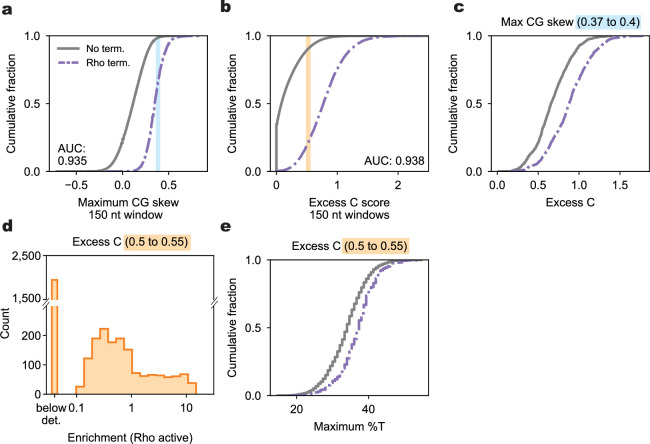
Performance of excess C and maximum %T scores. Related to Fig. 2. **a**, Cumulative distribution of maximum CG skew in 150 nt windows, separated by fragments (length ≥ 150 bp) with (dotted purple line; n = 9,983) and without (solid grey line; n = 136,728) Rho termination activity. AUC, area under ROC curve (Methods). Light blue shaded region indicates maximum CG skews considered in **c. b**, As in **a**, but for the excess C score, which is the sum of all positive CG skews in 150-nt windows tiling the fragment (Methods). Orange shaded region indicates excess C score of fragments shown in **d** and **e**. **c**, As in **b**, for fragments with a maximum CG score between 0.37 and 0.40 (region highlighted in blue in **a**). Non-terminated and Rho-terminated fragments with very similar maximum CG skews are separated by their excess C scores. **d**, Distribution of enrichments for fragments (length ≥ 150 bp) with an excess C score between 0.50 and 0.55. Below det., below detection (Methods). **e**, Cumulative distribution of maximum %T score for fragments with an excess C score between 0.50 and 0.55, separated by fragments with (dotted purple line; n = 9,983) and without (solid grey line; n = 136,728) Rho termination activity. Fragments with very similar excess C scores are separated by their maximum %T score. Source data

**Extended Data Fig. 4:**
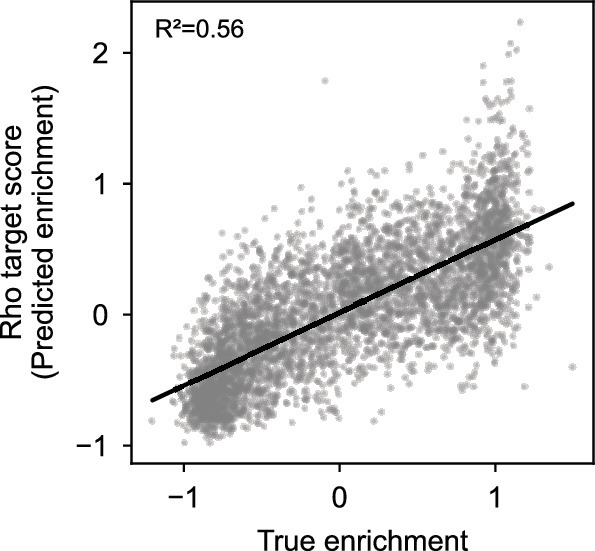
Multiple linear regression predicts enrichment from excess C and maximum %T scores. Related to Figs. 2, 3, 6. Scatterplot of predicted enrichment (Rho target score) vs. experimental enrichment for fragments retained in the test set (n = 3,958 fragments). Predicted and experimental enrichments are log_10_-transformed. Black line, linear regression between predicted and observed enrichment values (Methods). Enrichment was predicted by a linear model trained on the excess C and maximum %T values of 15,832 fragments split evenly into Rho-terminated and non-terminated fragments. All fragments in the test and training set were at least 150 nt in length. Pearson’s R^2^ value for the regression line shown at top left. Source data

Only 5% of all genomic fragments (Fig. 1d) (7% of antisense fragments, Fig. 2a) are terminated by Rho, further illustrating the high degree of sequence specificity in *B. subtilis* Rho’s target selection. We identified two distinguishing features of Rho-terminated fragments. First, many of them possess long stretches (>150 nt) of high C content relative to G (Extended Data Fig. 3a–c and Methods), consistent with previous observations of *E. coli* Rho’s sequence preference^5,16,22,25,27,36,37^. We developed a metric for excess C content that quantifies the cumulative CG bias in each fragment (Methods). Although fragments with high excess C values are nearly all terminated by Rho, those with intermediate values exhibit a wide range of termination activities (Fig. 2b and Extended Data Fig. 3d). We identified a second feature—thymine (T) richness—that distinguished Rho-terminated fragments with intermediate excess C scores (Fig. 2b and Extended Data Fig. 3e). Together, a linear combination of these two pyrimidine-favouring metrics (‘Rho target score’) separates the fragments by their termination activity, explaining 56% of the variability in enrichment (Fig. 2b and Extended Data Figs. 2b and 4).

**Extended Data Fig. 5:**
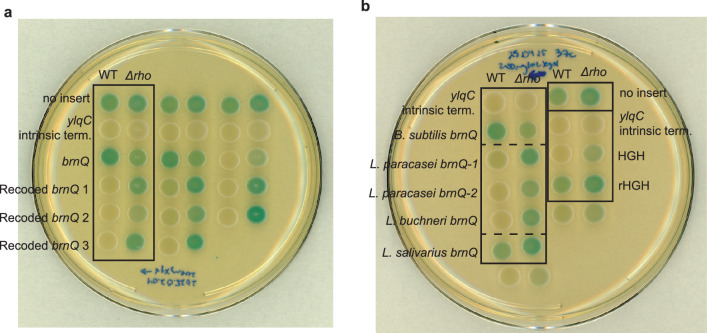
Uncropped X-gal plate images for LacZ assays. Related to Figs. 2, 3, 6. **a**, Full image for Fig. 2c. Black rectangle indicates cropping region. **b** Full image for Figs. 3d, 6a. Black rectangle indicates cropping region.

To show that the presence of pyrimidine-rich sequence features is sufficient to induce Rho termination in *B. subtilis*, we increased the Rho target score of a native gene that is not regulated by Rho (*brnQ*) by introducing synonymous mutations. All three recoded versions (5–8% mutation rate) suppressed expression of a downstream β-galactosidase gene (*lacZ*) included as a transcriptional reporter (Fig. 2c and Extended Data Fig. 5a). Deleting *rho* restored *lacZ* expression, indicating that recoding made these sequences targets of Rho-dependent termination (Fig. 2c). This result shows that the introduction of sequence signals recognized by Rho is sufficient to drive termination in *B. subtilis*.

**Extended Data Fig. 6:**
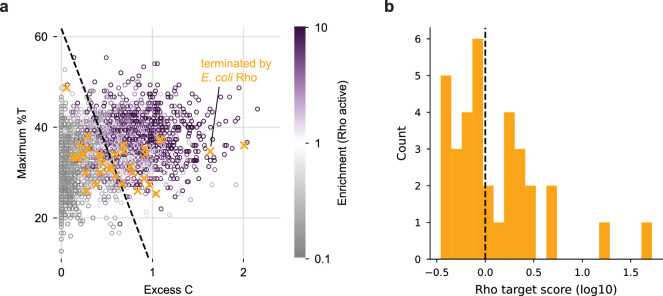
B. subtilis Rho is more selective than E. coli Rho. Related to Fig. 2. **a**, As in Fig. 2b, but overlaid with sequences strongly terminated by *E. coli* Rho *in vitro* (orange X)^37^. **b**, Distribution of Rho target scores for *E. coli* Rho-terminated sequences shown in **a**. Dashed line indicates enrichment cutoff of 1. Source data

Several lines of evidence indicate that the sequence requirement for *B. subtilis* Rho to terminate its RNAP is more stringent than that for *E. coli*. First, a well-established target of Rho termination in *E. coli*, *lacZ*^24,29,30,38,39^, is not subject to Rho-dependent termination in *B. subtilis* (Fig. 2c (no insert control) and Methods). Second, sequences that are known to be terminated by *E. coli* Rho in vitro^37^ do not have strong Rho target scores as we defined for *B. subtilis* (Extended Data Fig. 6a,b). Together, these results suggest that Rho termination occurs on a less diverse set of sequences in *B. subtilis* than in *E. coli*.

### Rho avoidance drives gene purine bias

**Fig. 3:**
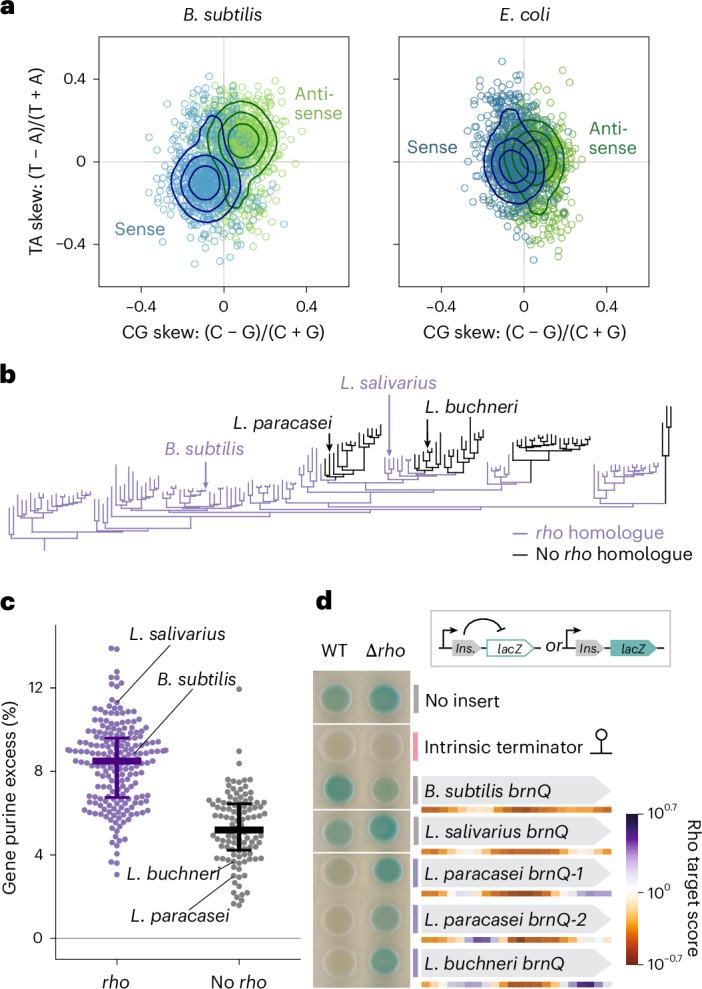
Rho avoidance drives purine bias in Bacilli genes. **a**, Nucleotide skew of 300-nt fragments of the *B. subtilis* and *E. coli* genomes derived from sense or antisense strands of genes (Methods). Each point represents a single fragment. Contour lines mark the 25th, 50th and 75th percentiles of Gaussian kernel density estimate. **b**, Phylogenetic tree of selected Bacilli species (156 species) coloured by presence or absence of a *rho* homologue in the genome^41^ (Methods). *L. paracasei*, *Lacticaseibacillus paracasei*; *L. salivarius*, *Ligilactobacillus salivarius*; *L. buchneri*, *Lentilactobacillus buchneri*. **c**, Average excess purine content in leading strand genes of 309 Bacilli species, separated by the presence (*n* = 198 species) or absence (*n* = 111 species) of *rho* in the genome (Methods). Purine excess quantifies the difference between purine and pyrimidine counts, normalized to length. The median and 25th and 75th percentiles are marked by horizontal lines. **d**, LacZ assays for termination of *brnQ* homologues in WT and Δ*rho B. subtilis*, as in Fig. 2c. Coloured bars summarize the result: grey, no termination; purple, Rho termination; and pink, Rho-independent termination. Insert sequences are available in Supplementary Table 3. An uncropped plate image is available in Extended Data Fig. 5b. Source data

Unlike the genes of other organisms, such as *E. coli*, we found that the coding strand of *B. subtilis* genes has strongly biased purine versus pyrimidine content (54% versus 46%; Figs. 2d and 3a). We hypothesized that this nucleotide bias is a result of selective pressure to avoid premature Rho termination of runaway RNAPs, analogous to the depletion of termination factor motifs in eukaryotic genes^40,41^. By the rules of base pairing, the complementary pyrimidine bias is then present in antisense transcripts (Fig. 3a), priming their suppression by Rho. Our hypothesis predicts that in the absence of Rho, the gene purine bias would be lost during evolution. Conveniently, several isolated Bacilli lineages have lost *rho*^42,43^ (Fig. 3b), and these *rho*-less species indeed exhibit a much weaker purine bias in their coding sequences (Fig. 3c). Furthermore, genes from *rho*-less species are prematurely terminated by Rho when expressed in *B. subtilis* (Fig. 3d and Extended Data Fig. 5b), confirming that the relaxed purine bias is accessible only in the absence of Rho. Therefore, for most Bacilli, the necessity to avoid Rho termination of runaway transcription is probably a major driver of the genome-wide separation between the nucleotide composition of sense and antisense strands.

**Fig. 4:**
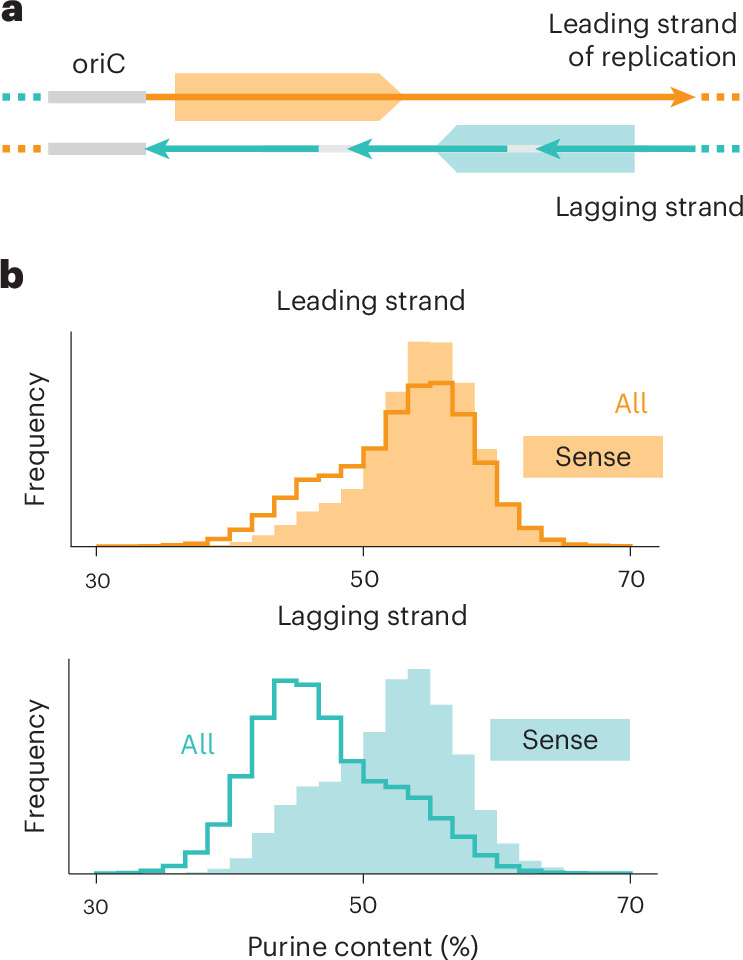
Purine skew is driven by the direction of transcription, not replication. **a**, Schematic illustrating regions considered in **b**. Thin arrows indicate the direction of replication. Thick arrows represent coding sequences positioned on each replication strand. oriC, origin of replication. **b**, Top: purine content of 300-nt sequence fragments derived from any position on the leading strand (all; thick orange line), or sense to genes (sense; filled) (Methods). Bottom: same as top, but for the lagging strand of replication. Source data

We next addressed a competing hypothesis that the purine bias arose from mutational biases in DNA replication. Bacillota genomes have purine-rich leading strands of replication and pyrimidine-rich lagging strands, which has been attributed to their possession of dedicated DNA polymerases for synthesis in each direction (PolC and DnaE)^44–46^. As most genes reside on the leading strand (74% in *B. subtilis*^46^), replication-based nucleotide bias would result in an overall purine bias in coding sequences. To decouple replication biases from the Rho-based selective pressure we propose, we analysed the purine–pyrimidine bias in lagging strand genes (Fig. 4a). Despite the overall depletion of purines on the lagging strand, coding sequences on this strand are purine rich, with the median purine content closely matching that of leading strand genes (53% versus 54%, respectively) (Fig. 4b). This and other evidence^47^ suggest that purine bias is a feature of genes, not the direction of DNA replication, consistent with a need to avoid Rho-dependent transcription termination.

### Purine bias illuminates a hidden code

**Fig. 5:**
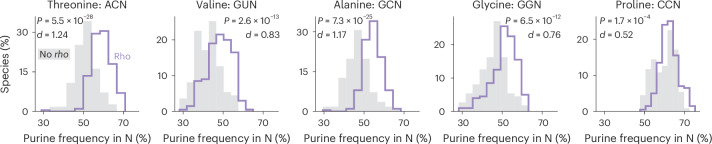
Rho shapes codon usage in Bacilli. Distribution of purine frequency in the wobble position among the codons used to encode the given amino acid across Bacilli species that do (thick purple lines; *n* = 200 species) and do not (filled grey bars; *n* = 111 species) encode *rho*^41^ (Methods). The five amino acids that are encoded by four codons are shown. N, any nucleotide. *d*, Cohen’s *d* effect size (Methods). *P* values from one-sided Mann–Whitney *U* tests. Source data

**Extended Data Fig. 7:**
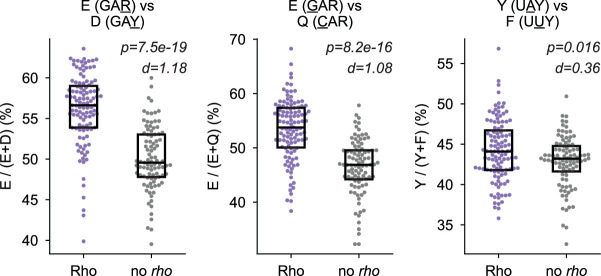
Rho shapes amino acid usage in Bacilli. Related to Fig. 3. Distribution of relative amino acid usage in Bacilli species separated by the presence or absence of a *rho* homolog^42^. Relative amino acid usage is shown for amino acid pairs with a strong ( ≥ 2) BLOSUM-62 substitution score^48^, where amino acid substitution requires a change in purine content in the nucleotide sequence without necessitating a change in GC content. Bacilli species shown have similar GC content (35–45%). n = 204 species (111 Rho, 93 no *rho*). Boxplots show median, 25^th^, 75^th^ percentiles. Underlined residue indicates position where purine content differs between amino acids. R, purine; Y, pyrimidine; N, any nucleotide. P-values from one-sided Mann-Whitney U tests. d indicates Cohen’s d effect size (Methods). Source data

Purine bias has the potential to break the redundancy in the genetic code, as many amino acids can be encoded by synonymous codons of differing purine content. We compared the codon usage for these amino acids between Bacilli species that have maintained and lost *rho*. Focusing on the amino acids with four codons, in which a purine-rich codon can be selected without necessitating a change in GC content, we found that codons that end with a purine are clearly favoured in species that encode Rho (Fig. 5). Furthermore, some amino acids have only codons with an identical number of purines but can be replaced by other similar amino acids to increase the purine content^48^. For several such pairs of permissive substitutions, we observed a preference towards purine-rich amino acids in Bacilli species with Rho (for example, glutamate with GAR codons versus aspartate with GAY codons), although these preferences may reflect multiple constraints on amino acid usage (Extended Data Fig. 7). These results show that the purine bias creates a hidden layer constraining the usage of the genetic code.

**Fig. 6:**
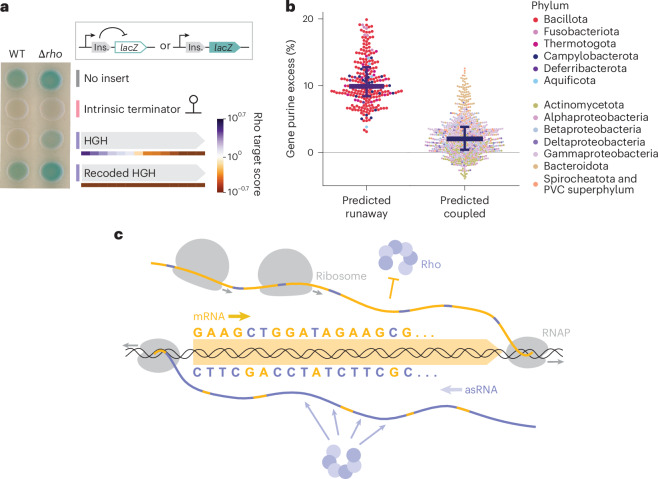
Purine-rich coding sequences protect runaway RNAPs from Rho. **a**, LacZ assays for termination of native and recoded HGH coding sequence in WT and Δ*rho B. subtilis*, as in Fig. 2c. Coloured bars summarize the results: grey, no termination; purple, Rho termination; pink, Rho-independent termination. Insert sequences are available in Supplementary Table 3. An uncropped plate image is available in Extended Data Fig. 5b. **b**, Average excess purine content in leading strand gene sequences across *n* = 1,002 bacterial genomes that encode Rho, separated by phylum-level prediction of runaway or coupled transcription^8^ (Methods). Purine excess quantifies the difference between purine and pyrimidine counts, normalized to length (Methods). The median and 25th and 75th percentiles are marked by horizontal lines. PVC, Planctomycetota, Verrucomicrobiota, and Chlamydiota. **c**, Schematic of sequence-based selection of Rho targets during runaway transcription. Purine-rich genes are protected by Rho’s pyrimidine preference, while base pairing dictates that the antisense strand is enriched for pyrimidines, promoting Rho termination. asRNA, antisense RNA. Source data

The hidden code imposed by Rho further creates a barrier for the expression of foreign genetic elements, such as bacteriophages and engineered constructs. To illustrate this barrier in the context of recombinant protein production, we show that transcription of a gene native to a different host (human growth hormone (HGH)) is terminated by Rho in *B. subtilis* (Fig. 6a and Extended Data Fig. 5b). Expression can be restored upon synonymous recoding to increase purine content. This example shows that foreign sequences that are not optimized to avoid Rho may not be compatible with runaway transcription in *B. subtilis*. This barrier could suppress heterologous protein expression and, analogous to *E. coli* Rho^49^, constrain horizontal gene transfer and protect the host from selfish elements, albeit through a different targeting mechanism.

Beyond Bacilli, this hidden code probably applies broadly to species across the bacterial tree that have runaway transcription, including both gram-positive and gram-negative bacteria. In contrast to species predicted to have coupled transcription–translation, the coding genomes of species predicted to exhibit runaway transcription^10^ are consistently biased towards purines (Fig. 6b). The strong prevalence of purine-rich coding sequences in these species suggests that evolutionarily distinct phyla depend on a common sequence-based protection to avoid premature Rho termination, have similar constraints on mRNA and protein sequences, and represent an alternative paradigm for transcriptional surveillance.

## Discussion

We discovered that many bacterial genomes have evolved strong purine bias across their coding sequences to escape termination during runaway transcription. This nucleotide code underpins the biased implementation of the genetic code, dramatically affecting gene expression levels and the fate of exogenous genetic elements. Meanwhile, antisense transcripts have an opposing pyrimidine bias that coincides with Rho’s heightened specificity, enabling Rho to maintain its function as a surveillance factor against spurious transcription^17,18,35^ (Fig. 6c). The broad separation of purines and pyrimidines into the coding and noncoding strands constitutes a new axis that shapes bacterial genomes, orthogonal to the well-appreciated GC–AT content.

The increased sequence specificity of Rho termination in *B. subtilis* compared with *E. coli* could be due to several factors, as this process involves both Rho and RNAP. First, the runaway RNAP in *B. subtilis* is nearly twice as fast as the *E. coli* RNAP^10,11^. A faster elongating RNAP could kinetically outcompete Rho, increasing the barrier to Rho termination^12–15,50^. Notably, *B. subtilis* RNAP pauses on T-rich sequences^51^, which could explain the increased Rho termination we observed for regions with high T content. Second, several *trans*-acting factors, including the transcription elongation factors NusG and NusA, nucleoid-associated proteins, sRNAs and direct Rho inhibitors, are known to modulate Rho activity in *E. coli*^12,14,16,22,23,39,52,53^*.* Deviations in the function of these proteins between *E. coli* and *B. subtilis* may also contribute to the differences in Rho specificity^51,54,55^. Finally, Rho termination requires both RNA-binding and ATP-dependent translocation^12–15,22,23,52^. Although the in vitro helicase activity of *B. subtilis* Rho exhibited a higher sequence stringency than *E. coli*^35^, this difference is not sufficient to explain the nearly complete depletion of Rho targets among coding sequences in vivo, suggesting that additional processes contribute to the stronger pyrimidine preference. Precisely mapping the exact positions of termination could help further dissect how sequence influences each step of termination.

Additional processes may also influence the purine content of genes. Even in the absence of runaway transcription or Rho, genes in many organisms still exhibit a mild purine bias, including *E. coli* (Fig. 3a) and Bacilli that have lost Rho (Fig. 3c). In *E. coli*, transient uncoupling of ribosomes may make the RNAP prone to Rho termination^4,56,57^, which could be mitigated by reducing the pyrimidine content. More broadly, any mutational biases in DNA replication could also contribute to a weak nucleotide preference in gene sequences^58,59^. Indeed, although *B. subtilis* genes on both the leading and lagging strands of replication exhibit a purine bias to counteract Rho termination, the bias is stronger on the leading strand (Fig. 4b). Finally, requirements for amino acids with certain chemical properties may influence purine content through the sequences of the corresponding codons. When runaway transcription arose, a mild pre-existing purine bias from any of these factors might have set the stage for coevolution of Rho’s heightened pyrimidine requirement and coding sequence composition.

Our study highlights that optimization of gene sequences is not merely defined by the effect of codon usage on translation—genes must adhere to requirements imposed by every step of gene expression. In bacteria, we show that a single transcription factor drives a profound coding constraint. We anticipate that systematic dissection of other central dogma processes will further unlock the principles for optimizing protein coding sequences in evolution and engineering.

## Methods

### Strains

*B. subtilis* strains were generated from 168 (bearing the *trpC2* mutation), and *E. coli* genomic DNA was extracted from MG1655. Linearized plasmids and genomic DNA were transformed using either standard protocols relying on natural competence^60^ or supercompetent *B. subtilis* strains bJD086 (xylose inducible, wild-type) and bJD087 (xylose inducible, Δ*rho*::*Kan*) based on ref. ^61^, or bCE017 (anhydrotetracycline (aTc) inducible, wildtype) and bCE019 (aTc inducible, Δ*rho*::*Kan*) based on ref. ^62^. bJD086 and bJD087 contain the xylose-inducible *comK* induction system from SCK6 (ref. ^61^) inserted at *amyE*. bCE017 and bCE019 contain the aTc-inducible *comK* system from (ref. ^62^) inserted at *amyE*.

Sequences of plasmids and integrations were confirmed by Sanger or nanopore sequencing. All strains are listed in Supplementary Table 2. All plasmids were generated in *E. coli* DH5-α cells using standard protocols and are listed with additional details in Supplementary Table 2.

### Genomes

The RefSeq^63^ entries for *B. subtilis* (NC_000964.3) and *E. coli* (NC_000913.3) were used to map the sequencing reads and generate genome fragments in silico, and the corresponding annotations were used to classify fragments overlapping with sequences sense or antisense to genes.

### List of oligonucleotides

Supplementary Table 2 contains the list of oligonucleotides used for strain construction and sequencing library generation.

### Supercompetent *B. subtilis* transformation

To clone individual *B. subtilis* strains and generate the *B. subtilis* reporter library, linearized plasmid was introduced into *B. subtilis* using an inducible competence system mediated by the xylose-inducible *comK* allele from ref. ^61^ or the aTc-inducible *comK* allele from ref. ^62^ integrated at the *amyE* locus. Briefly, a colony of bJD086 or bCE017 (wild-type background), or bJD087 or bCE019 (Δ*rho* background) was picked into LB and grown to OD_600_ 0.8–1.1 at 37 °C with vigorous shaking. At this point, 1% xylose (w/v) (for bJD086 and bJD087) or 10 ng ml^−1^ aTc (for bCE017 and bCE019) was added to induce expression of ComK. Induced cultures were shaken at 37 °C for an additional 1.25–2 h, at which point 200 ng linearized plasmid was added per 100 μl induced cells. The cells were cultured with DNA for an additional 1–1.5 h before plating on selective plates for overnight growth at 37 °C.

### Reporter plasmid library construction

The plasmid library was constructed through isothermal assembly of tagmented genomic DNA fragments into the reporter plasmid backbone, pJD16. To generate the inserts, a 1:1 mixture of *B. subtilis* and *E. coli* genomic DNA was fragmented by Nextera XT tagmentation (Illumina). Then, 6 ng of the tagmented genomic DNA was PCR amplified with oJD173/oJD174 for 10 cycles using Q5 DNA polymerase (New England Biolabs). PCR products 200–400 bp in length were size selected using an 8% TBE polyacrylamide gel (Thermo Fisher). This size-selected DNA was then cleaned up with a DNA Clean & Concentrator-5 column (Zymo Research). To linearize the pJD16 backbone, the pJD16 plasmid was PCR amplified with oJD091/oJD092 using Q5 DNA polymerase. The PCR product was extracted from an agarose gel using a Zymoclean Gel DNA Recovery Kit (Zymo Research) and DpnI (New England Biolabs) digested for 60 min at 37 °C. The DpnI-treated DNA was cleaned up with a DNA Clean & Concentrator-5 column.

To construct the plasmid pool, 200 ng of the pJD16 backbone PCR was mixed with insert at a 1:4 molar ratio and assembled at 50 °C for 15 min in a Gibson Assembly reaction (New England Biolabs). This reaction was then cleaned up with a DNA Clean & Concentrator-5 column and transformed into electrocompetent *E. coli* (New England Biolabs 10-β cells). Transformants were plated on LB with 100 μg ml^−1^ carbenicillin in 245-mm square bioassay dishes (Corning) and grown at 37 °C overnight. Cells were then scraped off the plates for ZymoPure II Maxiprep plasmid extraction (Zymo Research).

### Transformation and integration of reporter library into the *B. subtilis* genome

The supercompetent transformation protocol described above was used to integrate the reporter library into *B. subtilis* strain bJD086, replacing the *comK* induction cassette at the *amyE* locus. Briefly, a colony of bJD086 was picked into 22 ml LB and induced with 1% xylose (w/v) once the culture reached OD_600_ 1.02. After 1.5 h of growth in xylose, 15 ml of induced culture was combined with 1.5 ml (30 μg) of plasmid pool linearized with ScaI-HF (New England Biolabs). This digest was prepared according to the manufacturer’s protocol with 1 μg plasmid per 50 μl reaction. After 1.5 h of incubation with DNA, cells were pelleted, resuspended in residual media and plated on 245-mm bioassay dishes containing 100 μg ml^−1^ spectinomycin. After growth overnight, cells were scraped into LB with 100 μg ml^−1^ spectinomycin and approximately 500 million cells were back-diluted into LB with 100 μg ml^−1^ spectinomycin. This culture was grown for 5 h, and 1-ml aliquots of culture were mixed 1:1 with 40% glycerol and frozen at −80 °C.

### Library growth and collection

To perform chloramphenicol selection on the *B. subtilis* library to enrich for genomic fragments with transcription termination activity, the library was grown in LB for five generations, split into +BCM-Bz and DMSO control (−BCM-Bz) conditions for pre-treatment and then back-diluted into selective media containing chloramphenicol and BCM-Bz or chloramphenicol and DMSO. Additional details on the timing and OD_600_ measurements for this process are described in the Supplementary Methods. In total, cell pellets for four conditions were collected: +BCM-Bz culture before and after chloramphenicol selection, and −BCM-Bz culture before and after chloramphenicol selection.

### Genomic DNA sequencing

Sequencing libraries were prepared to quantify the frequency of each genomic fragment variant in the four experimental conditions. Genomic DNA was extracted from the cell pellets using the Wizard Genomic DNA Purification kit (Promega) following the manufacturer’s instructions. To reduce RNA contamination, the genomic DNA was then cleaned up with the Genomic DNA Clean & Concentrator-10 (Zymo Research). Libraries were generated using a two-step Q5 PCR protocol. To attach unique molecular identifiers, a two-cycle PCR was performed using 2 μg of genomic DNA per 150 μl PCR (scaled as needed for library complexity) with primers oJD180/oJD182. This PCR was cleaned up with two rounds of magnetic bead clean-up at a 1:1 ratio of beads to sample (PCRClean DX bead, Aline Biosciences). Half (preselection libraries) or one-quarter (post-selection libraries) of this cleaned-up reaction was then amplified in a second PCR with primers oJD181/oJD183. The second PCR was gel purified using an 8% TBE gel. These libraries were sequenced with 50-bp paired-end reads on a G4 sequencer (Singular Genomics).

### Fragment quantification

Quantification of genomic fragments in each sample was handled using custom Python scripts. First, the paired-end sequencing reads were aligned to the *B. subtilis* (NC_000964.3) and *E. coli* (NC_000913.3) genome using the bowtie package (version 1.2.3)^64^. Then, 40 nt of each read was used for alignment, and alignments with more than 1 mismatch or an insert size of more than 5,000 bp were discarded (bowtie arguments: -trim3 10 -v 1 -X 5000). Any two reads with the same aligned start and end positions and unique molecular identifier were collapsed to a single read. The number of unique reads mapping to each genomic fragment was calculated and normalized to the total number of reads in the sample. The enrichment of each fragment in each condition (+BCM-Bz or −BCM-Bz) is the ratio of its frequency in the post-selection sample to the corresponding preselection sample. Reads mapping to the *E. coli* genome were combined and used for normalization to total reads in the enrichment calculations, but were not included in further analysis. Raw read counts for all fragments and samples are available in Supplementary Table 4.

### Thresholding and pseudocounting enrichments

To reduce noise in the enrichment values, a threshold of ≥50 reads per fragment was applied to the preselection samples and a threshold of ≥25 reads per fragment was applied to the post-selection samples. Fragments that fell below the post-selection read threshold owing to depletion during selection were assigned a pseudo-enrichment value of 0.015 (labelled as below detection in Figs. 1d and 2a, and Extended Data Figs. 1 and 3d) provided that their raw enrichment was below 0.5 (−BCM-Bz) or 1 (+BCM-Bz). A total of 161,256 fragments passed these thresholds or were assigned a pseudo-enrichment in both conditions (+BCM-Bz and −BCM-Bz), and their raw read counts, normalized read counts and reported enrichments are available in Supplementary Table 4. In Figs. 1d and 2a, fragments with a pseudo-counted enrichment were assigned a random enrichment value to jitter points for visualization.

### Fragment classification

Two criteria were used to identify Rho-terminated fragments from their enrichment scores. First, fragments terminated by Rho should be enriched in the absence of BCM-Bz, when Rho is active. Rho-terminated fragments had to therefore exceed an enrichment of 1 in the −BCM-Bz condition, which roughly corresponds to ≥65% termination efficiency (Extended Data Fig. 1). Second, the enrichment observed in the absence of BCM-Bz should be lost when Rho is inactivated by BCM-Bz treatment. Thus, the enrichment of Rho-terminated fragments in the +BCM-Bz condition had to be more than twofold lower (0.45×) than the enrichment in −BCM-Bz. This cut-off was chosen to minimize misclassification of fragments with known intrinsic terminators as Rho-dependent termination sites (less than 1.6% misclassified). A total of 9,995 fragments were classified as driving Rho-dependent termination activity, the positions of which are available in Supplementary Table 4.

Fragments referred to as ‘non-terminated’ throughout the main text and figures were defined as those with an enrichment less than 1 in the −BCM condition. The positions of these fragments are identified in Supplementary Table 4. (Note that these fragments may include some cases of weak termination and are further separated in the classifications described below.)

Criteria for the additional classifications shown in Supplementary Fig. 1 and a discussion of potential limitations in fragment classification are provided in the Supplementary Methods.

### Fragment overlap with sense and antisense regions and intrinsic terminators

Fragments overlapping with sense and antisense regions of the *B. subtilis* genome were identified using the annotated RefSeq coding sequence (CDS) features for NC_000964.3. Sense and antisense fragments were required to fully overlap with the same or opposite strand of an annotated CDS, and any fragments that overlapped with multiple coding regions (on either strand) were discarded. In total, 47,550 sense and 54,692 antisense fragments of *B. subtilis* are shown in Fig. 2a. Fragments were annotated as containing an intrinsic terminator if any of the wild-type positions of intrinsic termination identified in ref. ^34^ fell within the fragment (*n* = 3,037 fragments). The termination efficiencies used in Extended Data Fig. 1 were determined from the readthrough fraction in Rend-seq of Δ*pnpA B. subtilis*^34^.

### Excess C and maximum %T metrics, linear model and Rho target score

To identify sequences with a skewed C content relative to G, we wanted to capture information about the magnitude of the difference between C and G counts in a defined region (representing a single Rho termination site) and also capture the cumulative effect of multiple regions where C content exceeds G content, which we hypothesized would increase the probability of Rho termination in the fragment. Both features are captured by the excess C score, which is described in detail in the Supplementary Methods.

Maximum %T was determined by counting the number of threonines (Ts) in every 150-nt window tiling a sequence fragment and then taking the maximum T count as a fraction of the window length. We found that the performance of metrics related to T content in distinguishing the Rho-terminated fragments was not improved by incorporating information about relative adenine content. Fragments shorter than 150 bp (2.1% of the input library) were excluded from sequence feature analysis.

To evaluate the explanatory power of the excess C and maximum %T metrics and predict enrichments in silico, a linear model was trained to predict fragment enrichments from these two scores. The ‘Rho target score’ describes the enrichment predicted by this model. The training process and coefficients for this model and corresponding boundary line are described in the Supplementary Methods.

### LacZ reporter strains

DNA fragments with potential Rho-dependent transcription termination sites were cloned upstream of *lacZ*, under the control of the IPTG-inducible pSpankHy promoter in the vector pJD19. Detailed methods describing the process to generate the LacZ strains are available in the Supplementary Methods.

For qualitative assays of β-galactosidase activity, blue–white screening was performed on X-gal (5-bromo-4-chloro-3-indolyl β-D-galactopyranoside) (GoldBio) plates. For each strain, a colony was picked into 5 ml LB and grown at 37 °C with vigorous shaking to OD 0.50–3.5. These cultures were then diluted to OD_600_ 0.005 in LB, and 3 μl of the dilutions were spotted onto LB-agar plates with 1 mM IPTG and 200 μg ml^−1^ X-gal. Plates were incubated at 37 °C overnight and imaged 16–17 h after plating.

The process for generating recoded *brnQ* and HGH sequences, and additional details on the *brnQ* homologue sequences, are provided in the Supplementary Methods.

### Rho homologue classification

For *Lentilactobacillus buchneri* and the genomes shown in Fig. 6b, classifications of genomes with and without a *rho* homologue were taken from ref. ^34^. For all other Bacilli species (Fig. 3c–e and Extended Data Fig. 7), the Rho homologue classifications in ref. ^42^ were used.

### In silico generation of *B. subtilis* and *E. coli* genome fragments

To analyse the nucleotide composition of sense and antisense sequences in the *B. subtilis* and *E. coli* genomes, 300-nt tiling fragments (step size: 50 nt) were generated from the sequence of all CDS features in the RefSeq annotation files. Features shorter than 300 nt were excluded. The antisense fragments were generated by taking the reverse complement of the sense sequence fragments. All fragments (*n* = 51,531 for *B. subtilis* and 57,477 for *E. coli*) were used to generate the Gaussian kernel density estimates shown in Fig. 3a, and a subset of 1,600 fragments, split evenly into sense and antisense strands, are shown in the scatter plot. For the unbiased leading and lagging strand fragments shown in Fig. 4, randomly selected start positions were used to generate 300-nt windows of genomic sequence.

For Fig. 4, the *B. subtilis* sense sequence fragments and randomly generated fragments were assigned to the leading or lagging strand based on their position relative to the origin (4,215,389 (ref. ^65^)), assuming replication arms of equal length.

### Bacilli phylogenetic tree

The phylogenetic tree of Bacilli (Fig. 3b) was generated from the Genome Taxonomy Database (GTDB) tree^66^ (version 226, downloaded June 2025). A total of 155 Bacilli species analysed in ref. ^42^ and *L. buchneri* were included in the tree, with Rho homologue classification based on species name using refs. ^34,42^ as described above. Only a subset of the 309 Bacilli species shown in Fig. 3c was included in this tree for visualization purposes. See the Fig. 3 source data for the full list of species included in the tree.

### Sense strand purine content in Bacilli and across Bacteria

To compare the purine content in sense sequences across Bacteria (Fig. 6b), we analysed a set of 1,551 RefSeq genomes with coupling predictions^10^ and an annotated origin of replication^67^. To correct for length variation across genes, the purine content was determined for a random 250-nt fragment of each annotated gene (genes shorter than 250 nt were discarded). Each gene was then assigned to the leading or lagging strand using the position of the origin of replication from ref. ^67^ and assuming replication arms of equal size. The average purine content was calculated separately for genes on the leading and lagging strands, and the leading strand averages for the 1,002 genomes that encode a *rho* homologue and were assigned to a named phylum in ref. ^10^ are shown in Fig. 6b. The designation of runaway versus coupled transcription was performed at the phylum level, based on ref. ^10^. Species in Bacillota, Fusobacteria, Thermotogota, Deferribacterota, Aquificota and Campylobacterota were classified as exhibiting runaway transcription. These data are available in the Fig. 6 source data.

The same process for analysis of sense sequence purine content was repeated for the 308 Bacilli genomes (GTDB classification of c__Bacilli) from ref. ^42^ and *L. buchneri* to generate the data for Fig. 3c.

### Codon usage

For each of the Bacilli genomes (GTDB classification) with a *rho* homologue assignment from ref. ^42^, the nucleotide sequences of the annotated gene sequences (CDS features) were downloaded from the National Center for Biotechnology Information (NCBI) RefSeq database^63^. The frequency of each codon across the entire coding genome was then normalized to the amino acid frequency to determine the codon usage by amino acid. To determine the frequency of codons with a purine in the third position, the frequency of codons ending with A and G were summed for each amino acid. For each amino acid considered, the purine frequency in the third codon position for each Bacilli species is available in the Fig. 5 source data.

### Extended data and supplementary figures

Methods related to all extended data and supplementary figures are provided in the Supplementary Methods.

### Statistics

To generate the *P* values reported in Fig. 5 and Extended Data Fig. 7, a one-sided Mann–Whitney *U* test was performed. Assuming underlying distributions of the same shape, the alternative hypothesis for the test was that the median relative frequency (of codon or amino acid usage, respectively) was higher for Bacilli with *rho* than for species without *rho*. The values for the *U* statistic are as follows: Fig. 5: T (19,385), V (16,585), A (18,872), G (16,243) and P (13,820), and Extended Data Fig. 7: E to D (8,853), E to Q (8,507) and Y to F (6,056). Cohen’s *d* was used to report the effect size of the difference in means (absolute difference between the means divided by pooled standard deviation) and is reported in the corresponding figure. The number of each species is available in the figure legend, and source data are provided.

For Extended Data Fig. 3a,b, the Area Under the Curve statistic was computed for the distribution of the maximum CG skew or the excess C score, respectively, in non-terminated fragments (*n* = 136,728 fragments) versus Rho-terminated fragments (*n* = 9,983 fragments). Fragments shorter than 150 nt were excluded from the analysis. Source data are provided.

A description of the calculation of the *R*^2^ statistic reported in the text and Extended Data Fig. 4 is available in the Supplementary Methods in ‘Linear model and Rho target score’.

### Reporting summary

Further information on research design is available in the Nature Portfolio Reporting Summary linked to this article.

## Supplementary information

Supplementary Information Supplementary Figs. 1 and 2 and Supplementary Notes.

Reporting Summary

Supplementary Table 1 Frequency of Rho termination of sense fragments in BCM-Bz and Δ*rho* libraries.

Supplementary Table 2 Strain, plasmid and oligo details.

Supplementary Table 3 Sequences used in LacZ assays.

Supplementary Table 4 BCM-Bz screen results and read counts, enrichments, classifications and sequence features for all fragments.

Supplementary Table 5 Δ*rho* screen results and read counts, enrichments, classifications and sequence features for all fragments.

## Source data

Source Data Fig. 1 Statistical source data.

Source Data Fig. 2 Statistical source data.

Source Data Fig. 3 Statistical source data.

Source Data Fig. 4 Statistical source data.

Source Data Fig. 5 Statistical source data.

Source Data Fig. 6 Statistical source data.

Source Data Extended Data Fig. 1 Statistical source data.

Source Data Extended Data Fig. 2 Statistical source data.

Source Data Extended Data Fig. 3 Statistical source data.

Source Data Extended Data Fig. 4 Statistical source data.

Source Data Extended Data Fig. 6 Statistical source data.

Source Data Extended Data Fig. 7 Statistical source data.

## Data Availability

All data generated or analysed during this study are included in the Article and Supplementary Information. The data discussed in this publication have been deposited in Gene Expression Omnibus of the NCBI^68^ and under Gene Expression Omnibus Series accession number GSE326227. Source data are provided with this paper.

## Code availability

The Python (version 3.11.7) scripts and notebooks used to calculate fragment enrichments (calculate_enrichment_pc.ipynb), assign termination status (assign_Rho_termination.ipynb), calculate sequence feature metrics (excessC_maxT_scores.py), compute the Rho target score (calculate_rho_target_score.ipynb) and calculate average purine excess in bacterial coding genomes (genome_process_script_pyr.py) are available via GitHub at https://github.com/jdierksheide/Rho (jdierksheide/Rho) with associated data and via Code Ocean at https://codeocean.com/capsule/6493993/tree.
