# Supplementary material for: Purine bias in bacterial genes is driven by runaway transcription
Source: Nat Microbiol. 2026 Jun 15;11(7):2065–75. doi: 10.1038/s41564-026-02389-1 (PMC13323073; doi:10.1038/s41564-026-02389-1)
Supplement: Supplementary file 1 — Supplementary Figs. 1 and 2 and Supplementary Notes. [file 41564_2026_2389_MOESM1_ESM.pdf]

nature microbiology

Supplementary information https://doi.org/10.1038/s41564-026-02389-1

Purine bias in bacterial genes is driven by runaway transcription

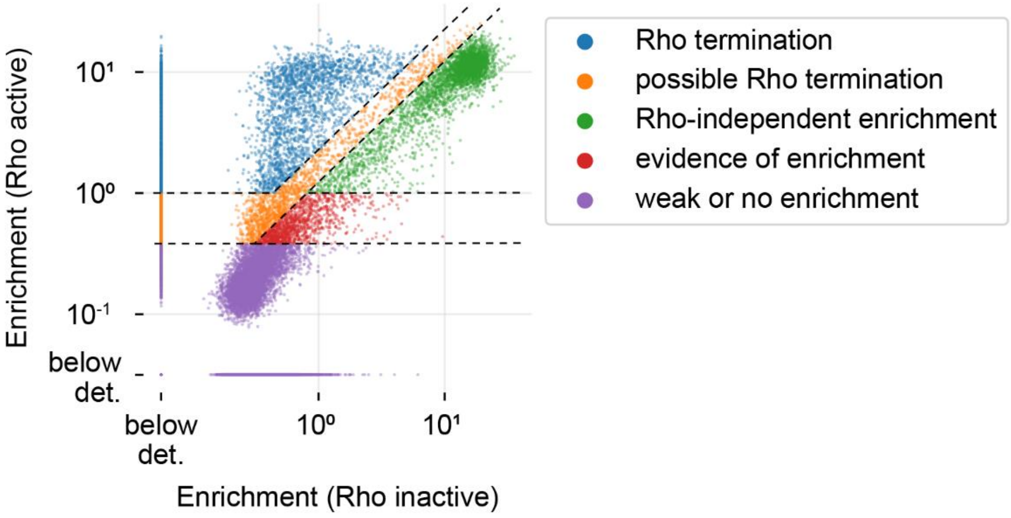

Supplementary Figure 1: Boundaries for fragment classifications. Enrichment of genomic fragments in the presence (Rho inactive) vs. absence (Rho active) of the Rho inhibitor BCM-Bz for a subset of 50,000 fragments. Color indicates fragment classification as Rho termination (blue), possible Rho termination (orange), Rho-independent enrichment (green), evidence of enrichment (red), or weak or no enrichment (purple). Dashed lines indicate the boundaries that separate each classification (Methods). Bet det., fragment below detection limit after selection (Methods).

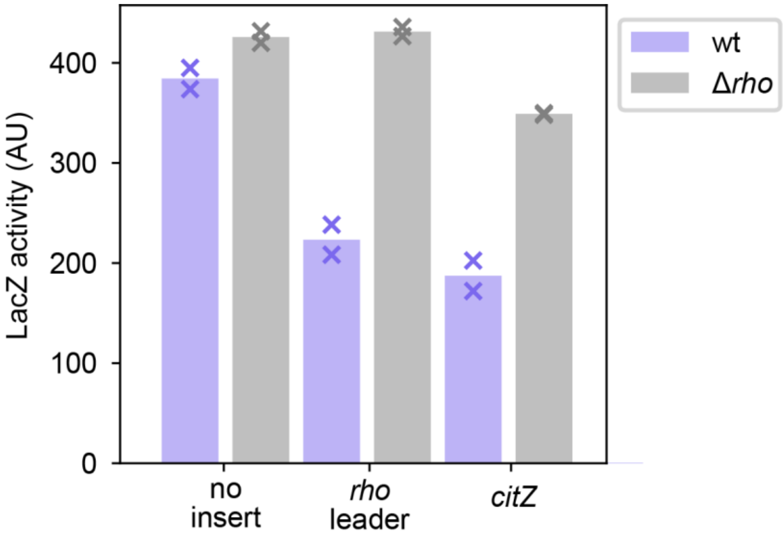

Supplementary Figure 2: Termination strength of known Rho terminators. Median β-galactosidase (LacZ) specific activity across 2-3 technical replicates when the indicated inserts are placed upstream of the *lacZ* gene in a wildtype (purple) or Δ*rho* (grey) background (Methods).

Supplementary Methods

Library growth and harvesting

To perform chloramphenicol selection on the *B. subtilis* library to enrich for genomic fragments with transcription termination activity, the library was grown in LB for 5 generations, split into +BCM-Bz and DMSO control (−BCM-Bz) conditions for pre-treatment, and then back-diluted into selective media containing chloramphenicol and BCM-Bz or chloramphenicol and DMSO.

To begin the initial outgrowth, a single glycerol stock was thawed, resuspended in warm LB, and back-diluted to OD_{600} 0.006 in 50 mL warm LB. This starter culture was grown at 37°C with vigorous shaking to OD_{600} 0.2. A second back-dilution to OD_{600} 0.006 in 25 mL was then performed to pre-treat the cultures with bicyclomycin-benzoate (BCM-Bz) or a DMSO control. For this pre-treatment, cells were grown either in LB with 120 µg/mL BCM-Bz (+BCM-Bz treatment) or 2.5% DMSO (−BCM-Bz treatment). The pre-treatment cultures were then grown to OD_{600} 0.2, at which point both cultures were harvested (pre-selection harvest). At the end of the +BCM-Bz pre-treatment, a small amount (3%) of residual chloramphenicol acetyltransferase is expected to remain in cells with inserts driving Rho termination, which may explain the slight increase in +BCM-Bz enrichment for these fragments relative to the non-terminated fragments (Fig. 1d). BCM-Bz concentration and pre-treatment duration were optimized using the *kinB* Rho termination site as a positive control. When the *kinB* coding sequence was placed in our reporter, cultures pretreated with BCM-Bz for 3 h exhibited a doubling time greater than 2 h in chloramphenicol, while the untreated control had a doubling time of 30 min.

To begin the chloramphenicol selection, the BCM-Bz pre-treated culture (+BCM-Bz) was back-diluted to OD_{600} 0.013 in 25 mL of LB with 5 µg/mL chloramphenicol and 120 µg/mL BCM, and the untreated culture (−BCM-Bz) was back-diluted to OD_{600} 0.0075 in 25 mL of LB with 5 µg/mL chloramphenicol and 2.5% DMSO**.** The cultures were then grown with vigorous

shaking at 37°C for 2.75 h before the post-selection harvest (OD_{600} 0.24–0.27). To harvest cells, 11–14 mL of culture was pelleted at 4°C and then frozen in liquid nitrogen. Cell pellets were stored at −80°C until gDNA extraction. In total, cell pellets for four conditions were collected: +BCM-Bz culture before and after chloramphenicol selection; −BCM-Bz culture before and after chloramphenicol selection.

Additional fragment classifications

In addition to the set of strong, high-confidence Rho termination sites, we relaxed both thresholds to highlight fragments with some evidence of Rho-dependent termination activity. These fragments with "possible Rho termination" had to exceed an enrichment of $10^{-0.42}$ in the −BCM-Bz condition and the ratio of the +BCM-Bz to −BCM-Bz enrichments had to be < 0.80.

Fragments with "Rho-independent enrichment" had to exceed an enrichment of 10^{0} in the −BCM-Bz condition (strong evidence of enrichment) and the ratio of the +BCM-Bz to −BCM-Bz enrichments had to be >0.80 (enriched independent of Rho inhibition). Fragments with "evidence of enrichment" exhibited an enrichment between $10^{-0.42}$ and 10^{0} in the −BCM-Bz condition (weakly enriched), and did not have a ratio of the +BCM-Bz to −BCM-Bz enrichments consistent with possible Rho termination. Finally, fragments with an enrichment below $10^{-0.42}$ in the −BCM-Bz condition were classified as having "weak or no enrichment" (based on the spread around the lower peak in the distribution of enrichments). These additional cutoffs are shown in Supplementary Figure 1 and the classifications for each fragment are available in Supplementary Table 4.

Limitations to fragment classification

We are aware of several limitations that may result in misclassification of Rho termination sites: (1) Rho termination sites in the genome may overlap with other cis-regulatory elements (such as

promoters or intrinsic terminators) that could influence enrichment in either condition and obscure the Rho-dependent termination activity, (2) we are likely to miss cases of weak Rho termination events, as fragments with weak termination activity are not consistently enriched (Extended Data Fig. 1), (3) we will not identify Rho termination sites that require more than ~300–400 bp for termination given the size distribution of our fragments, (4) we may miss Rho termination sites that are not fully contained within any of the fragments in our library due to incomplete coverage of all tiling windows.

In particular, we note that the lack of Rho-terminated fragments identified in the *rho* leader and *citZ* coding sequence, two regions with well-established Rho termination sites[1,2], is explained by the limitation in capturing weak termination events. In independent measurements of Rho termination strength, both regions exhibited a termination activity near 50% (Supplementary Fig. 2), which is not likely to drive strong enrichment in our screen (Extended Data Fig. 1).

Excess C score calculation

To identify sequences with a skewed cytosine (C) content relative to guanine (G), we wanted to capture information about the magnitude of the difference between C and G counts in a defined region (representing a single Rho termination site) and also capture the cumulative effect of multiple regions where C content exceeds G content, which we hypothesized would increase the probability of Rho termination in the fragment.

When we considered magnitude alone by identifying the highest CG skew ((C−G)/(C+G)) in windows tiling the sequence fragments, we found that a relatively short window (150 nt) best distinguished the Rho-terminated fragments (Extended Data Fig. 3a); averaging over longer sequences appeared to dilute the signal. We found that combining CG

skew across multiple windows further improved the prediction of Rho-terminated fragments. To capture the contribution of multiple windows with high CG skew, we summed the CG skew across all 150-nt tiling windows (step size: 50 nt) with positive CG skew (the number of Cs exceeds Gs). We refer to this metric as the "excess C" score (Extended Data Fig. 3b). A score of 0 indicates that in all 150-nt windows, the number of Gs matched or exceeded the number of Cs. We found that the cumulative information in the excess C score was important for predicting Rho termination: Rho-terminated and non-terminated sequences with similar maximum CG skews could be further differentiated by their excess C scores (Extended Data Fig. 3c). The window length, tiling step size, and skew cutoff were optimized for the highest predictive power in differentiating Rho-terminated and non-terminated fragments.

Linear model and Rho target score

To evaluate the explanatory power of the excess C and maximum %T metrics and predict enrichments in silico, a linear model was trained to predict fragment enrichments ($\log_{10}$-transformed) from these two scores. Fragments with enrichments below the detection limit were excluded from the test and training sets. The model was trained on 15,832 fragments (length ≥ 150 nt) split evenly into Rho-terminated and non-terminated fragments and the variance explained (Pearson R^2 value) was generated by evaluating the model on a reserved test set of 3,958 fragments (Extended Data Fig. 4). Fragments overlapping with 5′ ends identified by Rend-seq[3] were excluded to avoid confounding effects from promoter activity. The coefficients of the model are 0.0208 for maximum %T and 1.144 for the excess C score, and the intercept is −1.31. This linear model was also used to predict enrichments in silico from sequences' excess C and maximum %T scores, and these predicted enrichments are defined here as the "Rho target score." The R value reported in the text and Extended Data Fig. 4 corresponds to the Pearson's

correlation coefficient between the observed and predicted enrichments in the test set (n = 3,958 fragments).

The boundary line shown in Fig. 2a, c illustrates the positions on the plot where this Rho target score is 1 ($\log_{10}$-transformed enrichment of 0). Using our linear model, sequences that fall to the left of this line would be predicted to be depleted in our experiment, and sequences to the right would be predicted to be enriched due to Rho termination. The equation of the line is: maximum %T = −55.0 (excess C score) + 63.0.

LacZ reporter strain generation

DNA fragments with potential Rho-dependent transcription termination sites were cloned upstream of *lacZ,* under the control of the IPTG-inducible pSpankHy promoter in the vector pJD19. To generate pJD19, a restriction enzyme cloning site was introduced to pGJ02[4] and *lacZ-α* was replaced by a full-length *lacZ* allele optimized for expression in *B. subtilis. lacZ* was amplified from pESW830 (from E. Wirachman, *lacZ* sequence originally derived from pCAL1422[5]). This version of *lacZ* aligns to residues 45–3,075 of the *E. coli lacZ* sequence with 4 mismatches (full sequence is available in[4]). As *lacZ* Rho termination sites were identified between residues 140 and 425 in *E. coli*[6], we do not expect that the missing N-terminal sequence could explain the lack of termination in *B. subtilis.*

Recoded *brnQ* variants*,* native and recoded HGH sequences, and *brnQ* homologs were synthesized as Gene fragments (Twist Biosciences) and amplified by PCR, adding the native *brnQ* ribosome binding site (15 bp upstream of start codon) to drive translation. Wild-type *B. subtilis brnQ* and the *ylqC* intrinsic terminator control were amplified by PCR from gDNA. The sequence of each insert is available in Supplementary Table 3.

These PCR products were inserted into pJD19 by isothermal assembly or digested alongside pJD19 with EagI-HF and HindIII-HF (New England Biolabs) before ligation using QuickLigase (New England Biolabs). The resulting plasmids were then linearized by ScaI-HF (New England Biolabs) digestion and transformed into the *amyE* locus to replace the *comK* expression system in bJD086 (wild-type background) and bJD087 (Δ*rho::Kan* background) as previously described. For strains expressing wild-type or recoded versions of *B. subtilis brnQ,* the native copy of *brnQ* gene was then knocked out through transformation of gDNA from the Δ*brnQ::erm* strain following natural competence protocols.

Recoding *brnQ* and HGH

To increase the Rho target score of *brnQ,* synonymous mutations were introduced as follows. First, the codons corresponding to each amino acid were ranked first by the number of cytosine (C) and uracil (U) bases, and then by the number of adenine (A) bases. Codons with the highest C+U and A content were ranked highest so that codons with high pyrimidine content and low G content would be prioritized in the recoding. The three rare codons in *B. subtilis* (CUA, AUA, AGG)[7] were not included in the ranking. A subset of the residues in *brnQ* were then randomly selected for recoding. For each of these residues, the wild-type codon was replaced by the codon with the highest ranking for the corresponding amino acid (maintaining the wild-type codon if a higher rank could not be achieved). Recoded sequences with regions of high excess C and maximum %T content were chosen manually through comparison to the data in Fig. 2b. The sequence of each recoded variant is available in Supplementary Table 3.

For recoding of human growth hormone (HGH), the native sequence encoding the mature HGH peptide (generated after cleavage of signal peptide) was acquired from RefSeq (NM_000515.5). A similar procedure was then followed to remove pyrimidine-rich features

through synonymous mutation. For HGH, codons were ranked first by the number of cytosine (C) and uracil (U) bases, and then by the number of guanine (G) bases. Codons with the lowest C+U and highest G content were ranked highest, and rare codons were again removed. Recoding was performed as described above. A recoded sequence with minimal excess C and low maximum %T content was chosen manually through comparison to the data in Fig. 2b. The sequence of the native and recoded variants are available in Supplementary Table 3.

brnQ homologs in Rho and non-Rho species

brnQ homologs were identified by gene name in the RefSeq annotations for *Lacticaseibacillus paracasei* (NC_022112.1), *Lentilactobacillus buchneri* (NZ_CP073066.1), and *Ligilactobacillus salivarius* (NZ_CP117983.1). Protein sequence homology was verified by EMBOSS needle alignment[8]: reported similarities ranged from 49–55%. Sequences are available in Supplementary Table 3.

Relative amino acid usage

For each of the Bacilli genomes (GTDB classification) with a *rho* homolog assignment from[9], the nucleotide sequences of the annotated CDSs were downloaded from the NCBI RefSeq database[10]. To control for differences in GC content, a cohort of 204 species with similar GC content (35-45%) is shown. The frequency of each amino acid in the entire coding genome was determined as the sum of its codon frequencies. For amino acid pairs with a BLOSUM-62 score of 2 or greater[11], where substitution requires a change in purine content without necessitating a change in GC content (E to D, E to Q, Y to F), the frequency of the purine-rich amino acid was divided by the total frequency of both amino acids in the pair.

Plasmid construction for the Δ*rho* reporter libraries

The plasmid library for the Δ*rho* experiment was constructed through ligation of tagmented gDNA fragments into the reporter plasmid backbone pJD01. To generate the inserts, a 1:1 mixture of *B. subtilis* and *E. coli* gDNA was fragmented by Nextera XT tagmentation (Illumina) and PCR amplified with oEC001/oEC002. The PCR product and plasmid backbone were then digested with HindIII-HF and EagI-HF (New England Biolabs) for 60 minutes at 37°C. The digested PCR product and linearized backbone were then cleaned up with a DNA Clean & Concentrator-5 column (Zymo Research).

To construct the plasmid pool, 100 ng of linearized pJD01 was mixed with the digested insert at a 1:3 molar ratio and ligated at 25°C for 5 min in a Quick Ligase reaction (New England Biolabs). This reaction was then cleaned up with a DNA Clean & Concentrator-5 column and transformed into electrocompetent *E. coli* (New England Biolabs 10-β cells). Transformants were plated on LB with 100 μg/mL carbenicillin in 245 mm square bioassay dishes (Corning) and grown at 37°C overnight. Cells were then scraped off the plates for ZymoPure II Maxiprep plasmid extraction (Zymo research).

Transformation and integration of the Δ*rho* reporter library

The supercompetent transformation protocol was used to integrate the Δ*rho* reporter library into *B. subtilis* strains bCE017 and bCE019, replacing the *comK* induction cassette at the *amyE* locus. Briefly, colonies of bCE017 and bCE019 were picked into 32 mL and 34 mL LB, respectively, and induced with 10 ng/mL aTc once the cultures reached OD_{600} 0.95 and 1.06, respectively. After 2 h of growth in aTc, 30 mL of each induced culture was combined with 3 mL (60 μg) of Δ*rho* plasmid pool linearized with ScaI-HF (New England Biolabs). This digest was prepared per the manufacturer's protocol with 1 μg plasmid per 50 μL reaction. After 1.5 h of incubation with DNA, cells were pelleted, resuspended in residual media, and plated on 245 mm bioassay dishes

containing 100 μg/mL spectinomycin. After growth overnight, cells were scraped into LB with 100 μg/mL spectinomycin and approximately 500 million cells were back-diluted into LB with 100 μg/mL spectinomycin. This cultures were grown for approximately 6 h and 1 mL aliquots of culture were mixed 1:1 with 40% glycerol and frozen at −80°C.

Δ*rho* reporter library growth and harvesting

To perform chloramphenicol selection on the wild-type and Δ*rho B. subtilis* libraries to enrich for genomic fragments with transcription termination activity, the libraries were grown in LB for ~10 doublings then back-diluted into selective media containing chloramphenicol.

To begin the initial outgrowth, a single glycerol stock for each library was thawed and resuspended in 200 mL warm LB. These starter cultures were grown at 37°C with vigorous shaking to OD_{600} 0.2. The wild-type and Δ*rho* cultures were then back-diluted to OD_{600} 0.003 and 0.015 in 200 mL warm LB, respectively. Once the cultures reached OD_{600} 0.2, they were harvested (pre-selection harvest).

To begin the chloramphenicol selection, each culture was then back-diluted to OD_{600} 0.002 in 100 mL of LB with 5 μg/mL chloramphenicol. The cultures were then grown with vigorous shaking at 37°C for 2.5 h and then harvested (post-selection harvest). To harvest cells, 11–14 mL of culture was pelleted at 4°C and then frozen in liquid nitrogen. Cell pellets were stored at −80°C until gDNA extraction.

gDNA sequencing, Δ*rho* reporter libraries

Sequencing libraries were prepared to quantify the frequency of each genomic fragment variant in the four experimental conditions. gDNA was extracted from the cell pellets using the Wizard Genomic DNA Purification kit (Promega) following manufacturer’s instructions. Libraries were generated using a two-step Q5 PCR protocol. To attach UMIs, a 2 cycle PCR was performed

using 1.6 μg of gDNA per 100 μL PCR reaction (scaled as needed for library complexity) with primers oCEK071/oCEK083. This PCR reaction was then cleaned up with a DNA Clean & Concentrator-5 column, followed by a one-sided selection with a Select-a-Size DNA Clean & Concentrator MagBead Kit (Zymo Research) using a 260-bp cutoff, followed by a second clean up with the DNA Clean & Concentrator-5 column. This size-selected reaction was then amplified in a second PCR with primers oJT895/oCEK087. The second PCR reaction was concentrated using a DNA Clean & Concentrator-5 column, then gel-purified using an 8% TBE gel. These libraries were sequenced with 36-bp paired-end reads on a NextSeq500 (Illumina).

Fragment quantification, Δ*rho* reporter libraries

Quantification of genomic fragments in the wild-type and Δ*rho* backgrounds was handled as described for the BCM-Bz experiment in *Fragment quantification.* Raw read counts for all fragments and samples are available in Supplementary Table 5.

Thresholding and pseudocounting, Δ*rho* reporter libraries

To reduce noise in the enrichment values shown in Supplementary Table 1 and Extended Data Fig. 2b, a threshold of ≥20 reads per fragment was applied to the pre-selection samples and a threshold of ≥5 reads per fragment was applied to the post-selection samples. Fragments that fell below the post-selection read threshold due to depletion during selection were assigned a pseudo-enrichment value of 0.015 provided that their raw enrichment was below 0.5 (wild-type) or 1 (Δ*rho*). 5,146 fragments passed these thresholds or were assigned a pseudo-enrichment in both conditions (wild-type and Δ*rho*), and their reported enrichments are available in Supplementary Table 5.

Assignment of Rho-terminated fragments in Δ*rho* reporter library

Rho-terminated fragments were identified based on their enrichment in a wild-type and Δ*rho* background using similar criteria as the BCM-Bz experiment (see *Fragment Classification*). Briefly, Rho-terminated fragments had to exceed an enrichment of 1 in the wild-type condition and their enrichment in the Δ*rho* background had to be more than 2-fold lower (<40%) than the enrichment in the wild-type background. 469 fragments were classified as driving Rho-dependent termination activity, the positions of which are available in Supplementary Table 5.

Sequence features of fragments in Δ*rho* library

Overlap with sense and antisense regions and calculation of excess C and maximum %T metrics was performed as described above (see *Fragment overlap with sense and antisense regions and intrinisic terminators* and *Excess C and maximum %T metrics*). Fragments longer than 525 bp were excluded from the data shown in Extended Data Fig. 2b for consistency with the fragment sizes in the BCM-Bz fragment library.

Identifying genomic regions represented in both the Δ*rho* and BCM-Bz reporter libraries

To identify genomic regions represented by fragments in both the BCM-Bz and Δ*rho* reporter libraries for Extended Data Fig. 2a, each fragment in the BCM-Bz library was paired with the fragment in the Δ*rho* library with the highest overlap (length of overlapping sequence divided by the length of Δ*rho* fragment). These fragment pairs were then filtered for pairs where the degree of overlap for both the Δ*rho* fragment and BCM-Bz fragment (length of overlap divided by the length of corresponding fragment) exceeded 80%.

Using the Δ*rho* data, fragments with strongly Rho-dependent termination activity (Rho-terminated fragments with an enrichment < 1 in the Δ*rho* background) were identified, and any overlapping fragments in the BCM-Bz reporter library were identified from the above fragment pairings. These fragments (n = 184) are listed in the Source Data for Extended Data Fig. 2a.

Quantification of Rho termination in *rho* leader and *citZ* coding sequence

To measure the strength of Rho termination in the *rho* leader and *citZ* gene, both sequences were cloned upstream of *lacZ,* under the control of the IPTG-inducible pSpankHy promoter in the vector pEW62 (from E. Wirachman, *lacZ* sequence originally derived from pCAL1422[5]).

The *rho* leader (transcription start site to 342 nt into the coding sequence) and *citZ* gene (codons 2-373) were amplified by PCR from *B. subtilis* gDNA using oJD251/oJD252 and oJD255/oJD256, respectively. These PCR products were inserted into pEW62 by isothermal assembly using NEBuilder HiFi DNA assembly (New England Biolabs) to generate a linear product that was then transformed into the *amyE* locus to replace the *comK* expression system in bJD086 (wild-type background) and bJD087 (Δ*rho::Kan* background) as previously described.

For quantitative assays of β-galactosidase activity, cells were backdiluted to OD_{600} 0.0002 in LB with 1 mM IPTG and grown at 37°C with vigorous shaking. When the cultures reached OD_{600} 0.2, 1 mL aliquots of culture were pelleted at 4°C and stored at −80°C. To measure β-galactosidase specific activity, the cell pellets were washed once with Z buffer[12] and then resuspend in 1 mL Z buffer. The OD_{600} of the resuspension was measured for use in the calculation of specific activity. 500 µL of resuspended cells were then permeabilized with 7.5 µL of toluene and β-galactosidase specific activity was determined as described[12] after pelleting cell debris.

References

1. Ingham, C. J., Dennis, J. & Furneaux, P. A. Autogenous regulation of transcription termination factor Rho and the requirement for Nus factors in *Bacillus subtilis*. *Mol. Microbiol.* **31**, 651–663 (1999).
2. Delaleau, M. *et al.* Comprehensive mapping of transcription terminator Rho utilization (Rut) sites across the *Bacillus subtilis* genome. *Nucleic Acids Res.* **53**, gkaf765 (2025).
3. Lalanne, J.-B. *et al.* Evolutionary Convergence of Pathway-Specific Enzyme Expression Stoichiometry. *Cell* **173**, 749-761.e38 (2018).
4. Johnson, G. E., Lalanne, J.-B., Peters, M. L. & Li, G.-W. Functionally uncoupled transcription–translation in *Bacillus subtilis*. *Nature* **585**, 124–128 (2020).
5. Thomas, J., Lee, C. A. & Grossman, A. D. A conserved helicase processivity factor is needed for conjugation and replication of an integrative and conjugative element. *PLoS Genet.* **9**, e1003198 (2013).
6. Ruteshouser, E. C. & Richardson, J. P. Identification and characterization of transcription termination sites in the *Escherichia coli lacZ* gene. *J. Mol. Biol.* **208**, 23–43 (1989).
7. Moszer, I., Rocha, E. P. & Danchin, A. Codon usage and lateral gene transfer in *Bacillus subtilis*. *Curr. Opin. Microbiol.* **2**, 524–528 (1999).
8. Madeira, F. *et al.* The EMBL-EBI Job Dispatcher sequence analysis tools framework in 2024. *Nucleic Acids Res.* **52**, W521–W525 (2024).
9. Moreira, S. M., Chyou, T., Wade, J. T. & Brown, C. M. Diversification of the Rho transcription termination factor in bacteria. *Nucleic Acids Res.* **52**, 8979–8997 (2024).
10. O'Leary, N. A. *et al.* Reference sequence (RefSeq) database at NCBI: current status, taxonomic expansion, and functional annotation. *Nucleic Acids Res.* **44**, D733-745 (2016).
11. Henikoff, S. & Henikoff, J. G. Amino acid substitution matrices from protein blocks. *Proc. Natl. Acad. Sci. U. S. A.* **89**, 10915–10919 (1992).
12. Miller, J. H. *Experiments in Molecular Genetics*. (Cold Spring Harbor Laboratory, Cold Spring Harbor (N.Y.), 1972).
